# Management of compromised first permanent molars in a cohort of UK paediatric patients referred to hospital‐based services

**DOI:** 10.1111/ipd.12951

**Published:** 2022-04-15

**Authors:** Reem AlKhalaf, Aline de Almeida Neves, Fiona Warburton, Avijit Banerjee, Marie Therese Hosey

**Affiliations:** ^1^ Centre of Oral Clinical & Transitional Science Faculty of Dentistry, Oral and Craniofacial Sciences King's College London London UK; ^2^ Department of clinical dental sciences College of Dentistry Princess Nourah Bint Abdul Rahman University Riyadh Saudi Arabia; ^3^ Clinical Lecturer in Paediatric Dentistry Centre of Oral, Clinical and Translational Sciences Faculty of Dentistry, Oral and Craniofacial Science King's College London London UK; ^4^ Federal University of Rio de Janeiro Rio de Janeiro Brazil; ^5^ Oral Clinical Research Unit Faculty of Dentistry, Oral & Craniofacial Sciences King's College London London UK; ^6^ Restorative Dentistry Centre of Oral, Clinical and Translational Sciences Faculty of Dentistry, Oral and Craniofacial Science King's College London London UK; ^7^ Centre of Oral, Clinical and Translational Sciences Faculty of Dentistry, Oral and Craniofacial Science King's College London London UK

**Keywords:** caries, extraction, first permanent molar, general anaesthesia (GA), molar‐incisor hypomineralisation, paediatrics

## Abstract

**Background:**

There are diverse opinions among dentists about managing compromised first permanent molars (cFPMs) in children and a perceived lack of guidance to help them evaluate prognosis.

**Aim:**

To evaluate the current management of cFPM in children referred to a UK hospital centre and to report the severity of the affected teeth.

**Design:**

A service evaluation was undertaken, based on case records of medically fit children (6‐11 years) referred to for the management of cFPMs. The presence of hypomineralisation, post‐eruptive breakdown and the proposed care plans were recorded. Radiographic signs of severity were scored using the ICDAS index (intra/inter‐rater kappa 0.96/0.82).

**Results:**

From 349 records screened over a 4‐month period, 249 met the selection criteria. Almost 81% were planned to have extraction of at least one cFPM, whereas 19.3% were managed without extraction. More than half of the extraction cases (*n* = 105) had radiographic radiolucencies not exceeding the middle third of dentine in the worst‐affected FPM. At the time of extraction, the mean age of the patients was 9.8 years (±0.9). GA was used in 196 (97.5%) cases, and 40.8% had not received previous treatment in any of their cFPMs.

**Conclusion:**

Potentially restorable cFPMs in children is, most of the time, in a cohort of UK patients referred for tier 3 services, being managed by timed extractions under general anasethesia.

**Why this paper is important to paediatric dentists**

- To acknowledge the current management of cFPMs at both tooth and child levels within a specialist setting.
- To state the need for modern MI restorative strategies training to improve the clinical judgement of the prognosis of cFPMs and to manage more cases conservatively.
- To ultimately reduce the number of hospital admissions of GA for the extraction of permanent teeth in children.

## 1 INTRODUCTION

First permanent molars are the most caries‐prone teeth in children.^1^, ^2^, ^3^ These teeth are also known to be more prone to hypomineralisation enamel defects, affecting one of six children globally.^4^, ^5^ This places the clinician in a difficult position regarding their medium‐ to long‐term prognosis and to decide whether to extract or restore these compromised first permanent molars (cFPMs) in children younger than 11 years. Modern restorative biomaterials and techniques are helpful in selecting minimally invasive operative approaches.^6^ The timing of the clinical decision, however, is important at this age since opting to extract a tooth of ‘poor prognosis’ offers an opportunity for physiological space closure to occur. This is evident in the difference of opinions among dentists about managing cFPMs,^7^ which, in part, could be explained by the lack of contemporary restorative guidance to help clinicians judge the severity of damage and the prognosis of cFPMs.^8^, ^9^

A recent survey in the United Arab Emirates among General Dental Practitioners (GDPs) and specialists in paediatric dentistry found that almost 85% of them believed in restoring rather than extracting cFPMs.^10^ Similarly, 94% of Norwegian dentists and 74% of practitioners in France would choose to retain a cFPM.^11^, ^12^ Interestingly, a recent study into the cost‐effectiveness of different treatment options for hypomineralised FPMs within the German healthcare system has shown that, assuming spontaneous alignment occurs after extraction and no orthodontic intervention is needed, timed extractions of cFPMs are the best practice in the long term.^13^ Considering this scenario, and the other varying clinical scenarios, where not only hypomineralisation defects have to be taken care of and where malocclusion is a common situation, it is not surprising that disagreement between retaining and extracting cFPMs among paediatric dentists and orthodontists exists when mildly affected teeth are present, where the long‐term prognosis is not clear.^14^

UK clinical guidelines recommend extracting ‘poor prognosis’ cFPMs from children aged between 8 and 10 years to enable physiological space closure.^15^ This is a common reason for paediatric hospitalisation, since this treatment is often performed under general anaesthesia (GA).^2^ According to National Health Service England (NHSE) data, there were 42,911 extractions of multiple teeth in patients younger than 18 years in England in 2016/17, a 17% increase from 2012 and 2013. This has a significant impact on NHS health economics, with the total cost of these procedures since 2012 being approximately £165 m.^16^

The aim of this study was to assess how the specialist paediatric dental department at a London hospital NHS Trust managed children referred with cFPMs, following either an invasive extraction pathway or a minimally invasive restoration pathway.

## 2 MATERIALS AND METHODS

A service evaluation was conducted using the records of children who were referred to the paediatric dentistry unit at Guy's and St. Thomas' Hospitals NHS Foundation Trust (GSTT) for the evaluation and treatment of cFPM/cFPMs. Information obtained from patient care plans included specialist recordings of caries, hypomineralisation and post‐eruptive breakdown (PEB), as well as radiographs utilised to assess radiolucency (caries and/or PEB) severity. This study was approved by the GSTT Audit Committee as a service evaluation (reference no. 9569). All information disclosed in the study was kept confidential, and the hospital number of the participants was used to guarantee anonymity while data were securely stored.

### 2.1 Subjects

Patients included in this service evaluation were those referred to for the management of cFPMs in the period between January and April 2019. Participants were 6‐ to 11‐year‐old children (representative of children commonly undergoing this procedure in the UK) referred by general dental practitioners for the treatment of at least one cFPM. Data were obtained from the hospital records of 346 patients referred to the paediatric dental department at GSTT, through either the new patient clinic (NPC) or the joint orthodontic‐paediatric (JOP) clinic pathways, or those who were already care‐planned and scheduled for the extraction of one or more cFPMs (paediatric dental GA list) within the time frame of the study.

The inclusion criteria were children aged between 6 and 11 years (aligning with current UK orthodontic guidelines) with good diagnostic quality radiographs taken as part of their standard care/consultation, medically fit or only mildly asthmatic, and without any cognitive impairments. This service evaluation was conducted in two phases.

#### 2.1.1 Phase I (prospective assessment)

This patient cohort was referred by their local dental practitioners for the management of one or more cFPMs through either the NPC or the JOP clinic at GSTT, and their care was planned between January and April 2019. The patients in this cohort were seen at the clinic for a final diagnosis but were still waiting for the final decision on whether or not to have their cFPMs extracted. They were referred back to their local practitioners until they reached the probable chronological age for timed extractions when they would be reassessed at GSTT. The records were screened periodically for the final decision on the extraction until the first lockdown measures were enforced due to the COVID‐19 pandemic in England (March 2020). In August 2020, elective dental treatment was resumed at GSTT and records were reviewed again in September 2020.

#### 2.1.2 Phase II (retrospective assessment)

This was a cohort of patients who had undergone GA for the treatment of at least one cFPM at GSTT during the period of this service evaluation (January to April 2019).

### 2.2 Data collection

Data collected for all children were anonymised and included the following:

1. Total number of referrals due to cFPMs
2. Gender of the referred patients
3. Treatment outcomes:
  - Number of cases
    - number of children scheduled for (Phase I) or who had undergone treatment (Phase II),
    - number of non‐extraction cases who received restorative treatment in at least one cFPM on‐site or were referred back to their dental practitioners for restorative treatment,
    - number of children referred back to their primary care dental practitioners for ongoing review, probably to be assessed again in future and
    - number of children scheduled for or had extraction of at least one cFPM during the assessment period;
  - Patients' age
    - mean age of the patients who had not had or planned to have extraction of any of their cFPMs and
    - mean age of the patients at the moment of extraction;
  - Mode of anaesthesia used in extraction, that is, general anaesthesia (GA), inhalation sedation plus local anaesthesia or local anaesthesia alone;
  - number of FPMs extracted;
  - restorative/stabilisation prior to extraction
    - number of children indicated for FPM restorative stabilisation prior to extraction and
    - number of children who had not received restorative stabilisation prior to extraction; and
  - The average time between the diagnosis and scheduled extraction.
4. The presence of caries and/or enamel defects, with or without PEB in extraction cases (as diagnosed by the hospital practitioner at the time of the consultation):
  - number of children with any enamel defect (opacities or PEB) or caries on at least one FPM
5. Condition of the cFPMs
  - condition of the worst‐affected cFPM (caries and/or severity of MIH defect—mild or severe, with or without PEB).
  - condition of the extracted or scheduled for extraction FPMs (caries and/or severity of MIH defect—mild or severe, with or without PEB).
6. Radiographic signs of the severity of the worst‐affected FPM in each case of:
  - non‐extraction,
  - those scheduled for or undergoing extraction,
  - those sent for stabilisation prior to extraction and
  - those who never had stabilisation prior to extraction.
7. Radiographic signs of the severity of the extracted or scheduled for the extraction of FPMs.

### 2.3 Radiographic scoring

Trained and calibrated investigators (RA and AN) used the most recently available radiographic record to score each child's FPM using the radiographic criteria described in the International Caries Detection and Assessment System (ICDAS)^17^ and the International Caries Classification and Management System (ICCMS™).^18^ The radiographic ICDAS scoring system is comprised of seven grades: 0—sound surface; 1—radiolucency restricted to the outer half of the enamel; 2—radiolucency restricted to the inner half of the enamel; 3—radiolucency restricted on the outer third of dentine; 4—radiolucency restricted to the middle third of dentine; 5—radiolucency restricted to the inner third of dentine (usually linked clinically to ‘cavitation’); and 6—radiolucency reaching the pulp. The merged ICCMS radiographic scoring has four grades: 0 represents sound surfaces, whereas the ICDAS scores 1, 2 and 3 are combined as *initial* caries, the ICDAS score 4 represents *moderate* caries, and the ICDAS scores 5 and 6 represent *extensive* caries.

For scoring the radiolucency (caries and/or PEB severity) in enamel, the radiolucency was divided into the inner and outer enamel along the direction of the lesion. For scoring dentine lesions, the dentine between the advancing front of the radiolucency along the shortest distance and the pulp chamber was considered, thus dividing the dentine lesion depth into thirds. If a restoration was present, the tooth was scored only if there was secondary caries diagnosed (CARS—caries associated with restorations/sealants). A 1.5‐hr calibration session was conducted in a computer laboratory. The analysis was undertaken using a Dell 25‐inch LED monitor with 2560x1440 pixel resolution, using ROMEXIS software (version 4.51.R; Planmeca OY, Helsinki, Finland). The brightness of the screen was set at 60 degrees, and the colour control was set in natural mode. The agreement of and between the two researchers was tested 1 month after the calibration session. Based on a random selection of 40 patient files, the radiographic lesion depth score was recorded by the main researcher (RA) with an intra‐examiner agreement kappa value of 0.96. Inter‐examiner agreement was tested after scoring the randomly selected patients' files by the main researcher and the second investigator (RA and AN). The kappa value for the inter‐researcher agreement was 0.82, demonstrating almost a perfect agreement.^19^

### 2.4 Statistical analysis

Data were entered into a Microsoft Access database and imported into the Statistical Package for the Social Sciences (IBM SPSS Statistics for Windows, version 23.0; Armonk, NY: IBM Corp). All data were anonymised, and descriptive statistical summaries of all variables were used and are presented in text, figures and tables. A single‐variable logistic regression analysis and odds ratio analysis were performed to find out which independent variable is independently associated with restoration receiving/not receiving restoration/temporary treatment before extraction.

## 3 RESULTS

**FIGURE 1:**
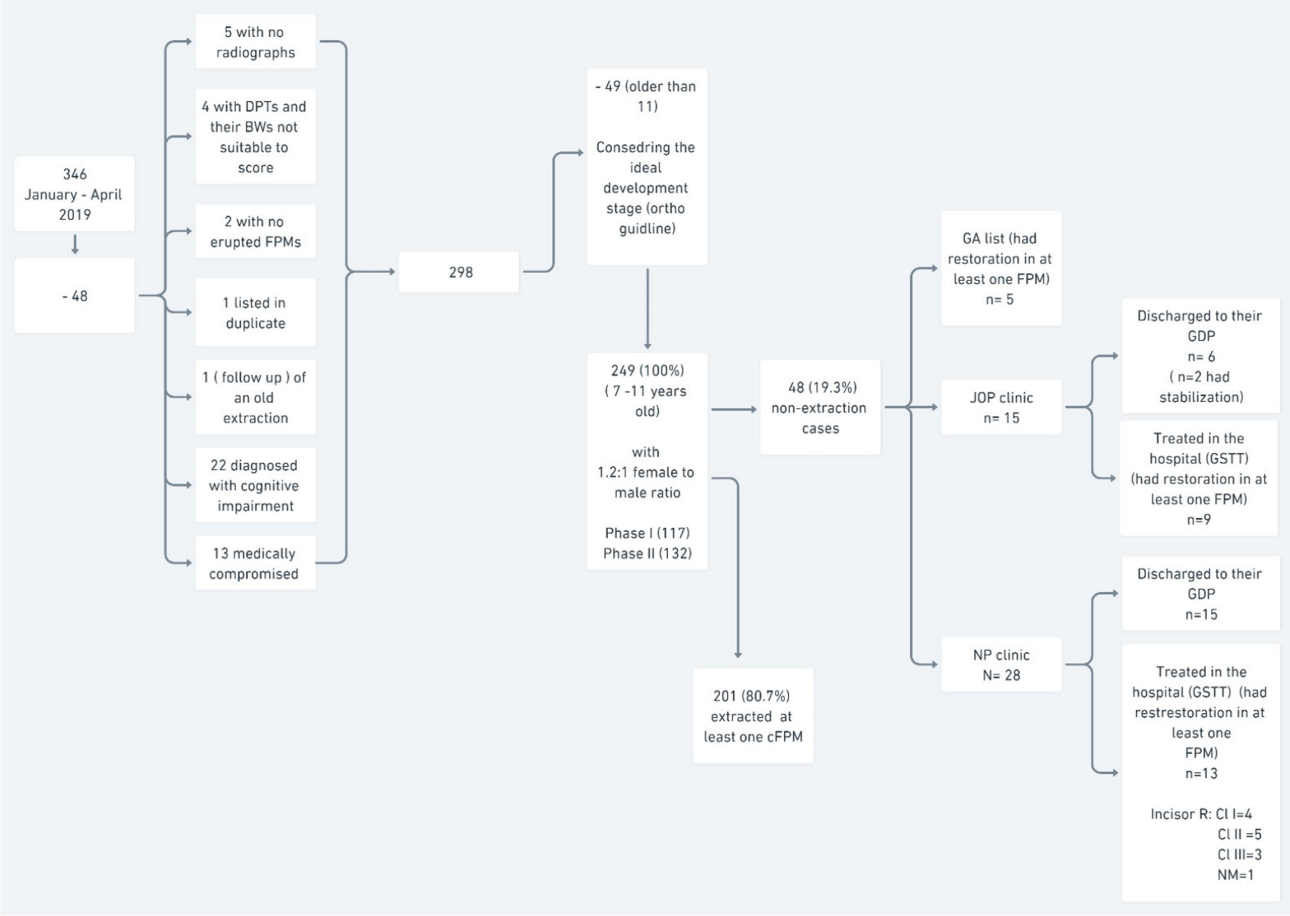
Summary and description of the total number of patient records analysed, including a detailed description of the fate of non‐extraction cases at the patient level

Case notes of 346 consecutive patients who attended the targeted clinical services were screened as part of this 4‐month service evaluation. After applying the inclusion criteria, 249 records were selected. One hundred and thirty‐four (53.8%) were female, and 115 (46.2%) were male (1.2:1 female‐to‐male ratio). Details of the sample selection process are shown in Figure 1. From the selected records, Phase I collection included 73 (29.3%) records of patients who attended the JOP clinic and 44 (17.7%) attending NPC during the study period, whereas 132 (53%) were identified during Phase II (patients who had undergone GA during the capture period).

**FIGURE 2:**
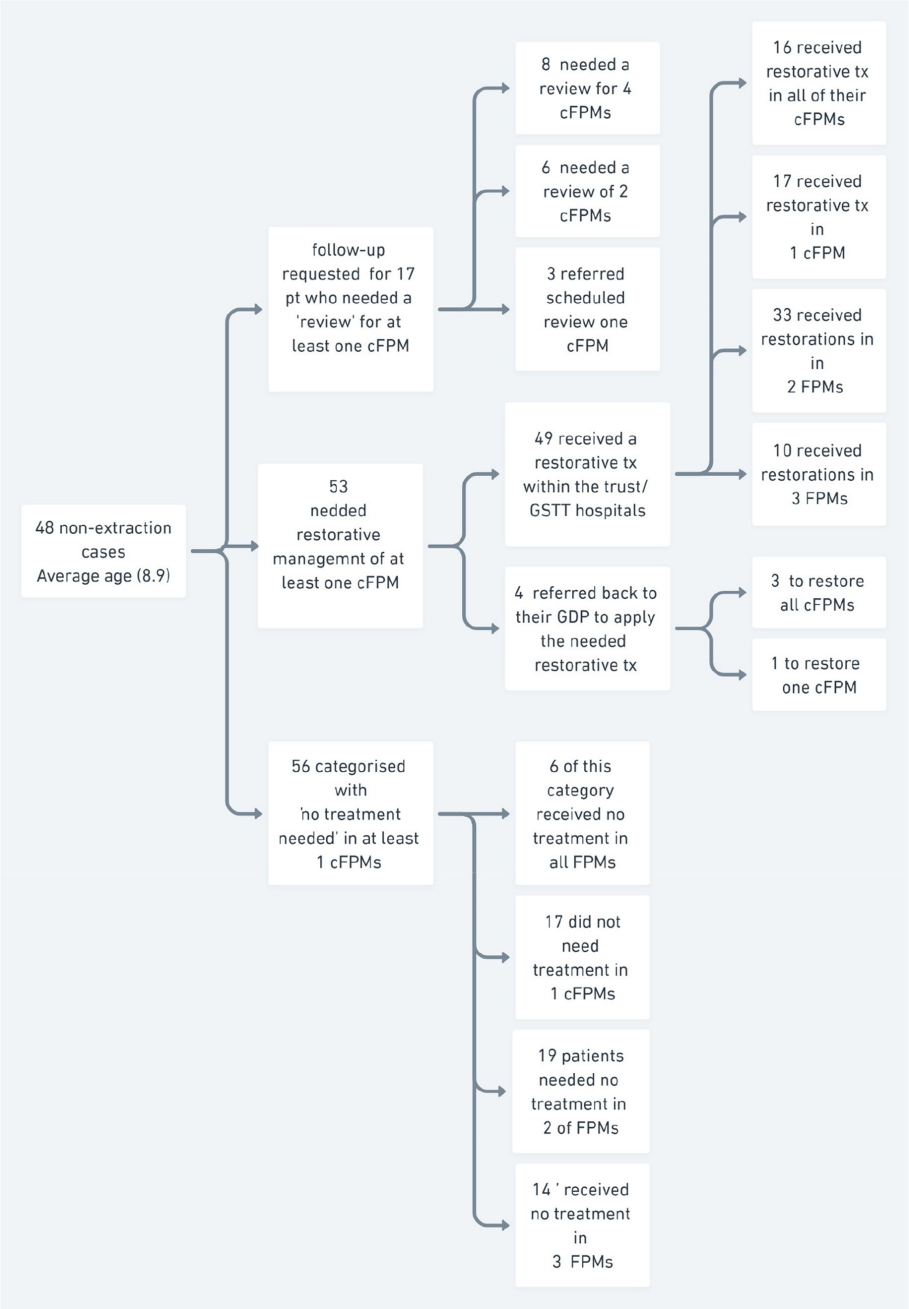
Description of non‐extraction cases at the tooth level

From the total sampled records (*n* = 249), 201 children (80.7%) were planned for at least one cFPM extraction, from which 74 were sampled in Phase I. Forty‐eight children (19.3%) either were planned for restoration and review or were deemed to have no treatment required in any of the cFPMs (non‐extraction cases, of which 43 were sampled in Phase I, and 5 in Phase II). A detailed description of non‐extraction cases at the tooth level is shown in Figure 2, and their distribution according to the severity of worst‐affected cFPMs (ICCMS, radiographic ICDAS) is detailed in Table 1. From the 117 children in Phase I, those who were planned for restoration and review or had no treatment required are detailed in Figure 1.

**TABLE 1:** Distribution of non‐extraction patients (*n* = 48) according to the severity of involvement of the worst‐affected cFPM

Radiographic ICDAS score of the worst‐affected FPM	ICCMS score of the worst‐affected FPM	Patients (*N*)	
0	Sound	35.4% (17)	35.4% (17)
RA 1	Initial	20.8% (10)	47.9% (23)
RA 2		16.7% (8)	
RA 3		10.4% (5)	
RB 4	Moderate	10.4% (5)	10.4% (5)
RC 5	Extensive	4.2% (2)	4.2% (2)
RC 6		0% (0)	
Restored/sealed	–	2.1% (1)	2.1% (1)
Total		100% (48)	

To identify the trigger for extraction, further data analysis concentrated on the 201 children who were planned for or had extractions of at least one cFPM in the combined sample (Phase I, 74 of 117 and Phase II, 127 of 132). The mean age of the patients at the moment of extraction was 9.8 ± 0.9 years (6.8–11.8 yrs). GA extraction was performed in 97.5% (196) of the children, local anaesthesia (LA) with inhalation sedation was used in 1.9% of the cases (4), and LA alone was used only for one case, as detailed in Table 2. In extraction cases, 127 were sampled in Phase I (69 used GA as a method of pain control during the extraction, 4 used inhalation sedation, and only one used LA alone), whereas 117 were sampled in Phase II (GA was used as a method of pain control). Regarding the number of cFPMs extracted per patient, 59.7% of children (120/201) had all FPMs removed, 8.5% (17) had three FPMs removed, 22.9% (46) had extraction of two FPMs, and 8.9% (18) had extraction of only one FPM.

**TABLE 2:** Distribution of extraction patients according to the number of cFPMs extracted, and mode of anaesthesia used

Number of extracted FPMs						
Mode of anaesthesia	One FPM	Two FPMs	Three FPMs	Four FPMs	Total number of patients (%) sampled in each phase	Total patients (%)
GA	15 (83.3%)	45 (97.8%)	17 (100%)	119 (99.2%)	69 (34.4%) (Phase I); 127 (63.2%) (Phase II)	196 (97.5%)
Sedation	3 (16.7%)	1 (2.2%)	0 (0%)	0 (0%)	4 (1.9%) (Phase I)	4 (2%)
Local anaesthesia alone	0 (0%)	0 (0%)	0 (0%)	1 (0.8%)	1 (0.5%) (Phase I)	1 (0.5%)
Total	18 (100%)	46 (100%)	17 (100%)	120 (100%)	201 (100%)	201 (100%)
Total number of patients sampled in each phase	7 (Phase I) 11 (Phase II)	16 (Phase I) 30 (Phase II)	6 (Phase I) 11 (Phase II)	31 (Phase I) 89 (Phase II)		

From the total number of patients who required/had extractions, 40.8% (82) had not received previous treatment in any of their cFPMs, whereas 59.2% (119) received some interim treatment, including glass‐ionomer cement (GIC) restorations in 20 children (16.8%), sealants in 13 children (10.9%) and resin composite restorations in 10 children (8.4%), 2 (1.7%) had dental amalgams placed, and 2 (1.7%) received stainless steel crowns (SSCs). Seventy‐two (60.5%) children received stabilisation without the specification of material and/or technique.

Distribution of patients according to the severity of worst‐affected cFPMs due to extraction (radiographic ICDAS and ICCMS), who had not received restorative stabilisation before extraction, showed that 61% (50 of 82) had a radiographic radiolucency reaching the middle third of dentine or deeper (RB4, RC5 and RC6) in at least one cFPM, as detailed in Table 3. This table also shows a statistically significant relationship between the ICDAS scores 3, 4 and 5 and the chances of receiving a restoration in at least one of the cFPMs before extraction (*p* = .023, .006 and .048 for the ICDAS scores 3, 4 and 5, respectively).

**TABLE 3:** Distribution of patients according to radiographic signs of severity of the worst‐affected FPM and status of tooth regarding temporisation before extraction (data in bold indicate statistical significance)

Radiographic ICDAS score of the worst‐affected FPM	ICCMS score of the worst‐affected FPM	Total extraction patients (*n* = 201)	No restoration received before extraction (*n* = 82)	Restored at least one cFPM before extraction (*n* = 119)	Odds ratio	95% CI	*p*‐value
0	Sound	10% (20)	15.8% (13)	5.9% (7)			.108
RA 1	Initial	4% (8)	4.9% (4)	3.4% (4)	1.857	0.352–9.795	.466
RA 2		5.5% (11)	4.9% (4)	5.9% (7)	3.250	0.701–15.071	.132
RA 3		16.9% (34)	13.4% (11)	19.3% (23)	3.883	1.210–12.467	.**023**
RB 4	Moderate	15.9% (32)	9.8% (8)	20.1% (24)	5.571	1.647–18.841	.**006**
RC 5	Extensive	19.9% (40)	18.3% (15)	21% (25)	3.095	1.010–9.485	.**048**
RC 6		27.8% (56)	32.9% (27)	24.4% (29)	1.995	0.693–5.745	.201

More than half of the extraction cases (105 patients, 52.3%) had a maximum radiographic radiolucency severity score of 4 (radiolucency reaching the middle third of dentine) in the worst‐affected cFPM, as detailed in Table 4. If only initial lesions (radiographic scores 0‐3) were counted, 36.4% of the patients who had at least one FPM extracted had the worst‐affected element presenting only with initial radiographic signs of defects.

**TABLE 4:** Distribution of patients according to the caries severity and the presence of enamel defects ± post‐eruptive breakdown (PEB) in the worst‐affected cFPM extracted

Radiographic ICDAS score of the worst‐affected FPM	ICCMS caries score of the worst‐affected FPM	Patients (*N*)		Hypomineralisation defects	PEB
0	Sound	10% (20)	10% (20)	9.4% (19)	5.8% (9)
RA 1	Initial	4% (8)	26.4% (53)	2.9% (6)	1.9% (3)
RA 2		5.5% (11)		5.4% (11)	4.5% (7)
RA 3		16.9% (34)		15.4% (31)	11% (17)
RB 4	Moderate	15.9% (32)	15.9% (32)	12.9% (26)	8.4% (13)
RC 5	Extensive	19.9% (40)	47.8% (96)	14.9% (30)	10.3% (16)
RC 6		27.8% (56)		15.4% (31)	7.1% (11)
Total		100% (201)		76.6% (154)^a^	49.3% (76)

^a^ In 23.4% of patients, the worst‐affected tooth had dental caries but did not have an enamel defect.

**FIGURE 3:**
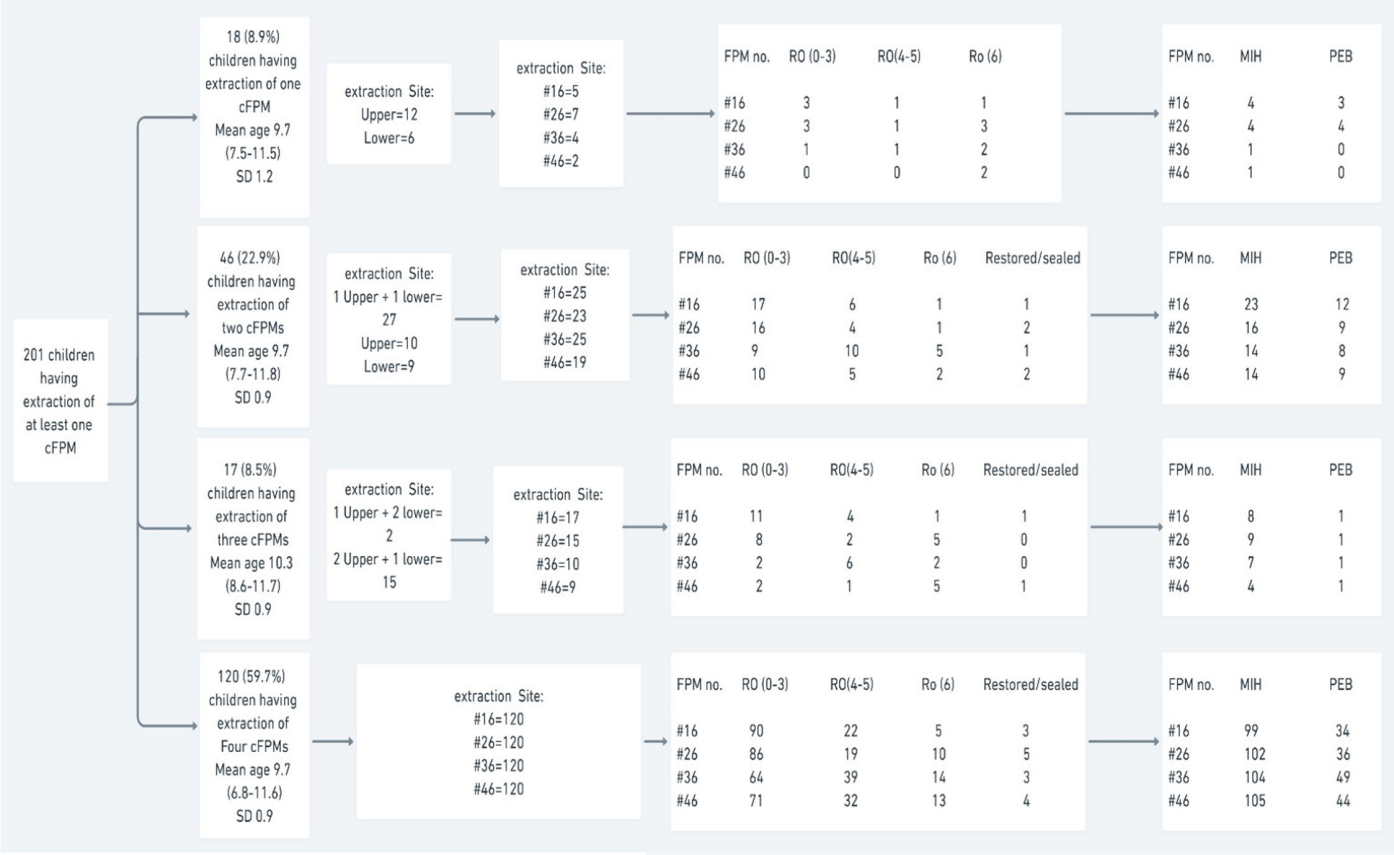
Description of extraction cases at the tooth level

Although 80.3% of extracted teeth had hypomineralisation defects, only 33% were diagnosed with PEB by the clinician (Table 5). More than 21% (137) of the extracted teeth were diagnosed clinically as sound regarding caries, showing only hypomineralisation‐related PEB, which appeared in radiographs as radiolucent areas (detailed in Table 5). Knowing that marked PEB and caries appear on the radiograph as a radiolucency, the assessment of the radiographs of each extracted tooth showed that over half of the extracted teeth (61.3%) had shallow lesions that did not exceed the outer third of dentine (further detailed in Table 5 and Figure 3). The specialists had not determined the final management of at least one cFPM in 20 (8%) of the assessed children.

**TABLE 5:** Description of extraction cases at the tooth level, a detailed description of each extracted or planned‐to‐be‐extracted FPM

Number of extracted FPM/s	RO (0–3)	RO (4–5)	RO (6)	Sealed	Total FPMs	Hypomineralisation defects	PEB	Sound with PEB clinically and RO^a^ radiolucency	RO^a^ scores of sound teeth (only PEB clinically)		
									RO (0–3)	RO (4–5)	RO (6)
1	7 (38.8%)	3 (16.6%)	8 (44.4%)	0 (0%)	18 (100%)	10 (55.5%)	7 (38.8%)	3 (16.6%)	3 (16.6%)	0 (0%)	0 (0%)
2	52 (56.5%)	25 (27.2%)	9 (9.8%)	6 (6.5%)	92 (100%)	67 (72.8%)	38 (41.3%)	28 (30.4%)	22 (23.9%)	6 (6.5%)	0 (0%)
3	23 (45.1%)	13 (25.5%)	13 (25.5%)	2 (3.9%)	51 (100%)	28 (54.9%)	4 (4.3%)	1 (1.9%)	1 (1.9%)	0 (0%)	0 (0%)
4	311 (64.8%)	112 (23.3%)	42 (25.5%)	15 (3.1%)	480 (100%)	410 (85.4%)	163 (33.9%)	105 (21.8%)	84 (17.5%)	20 (4.1%)	1 (0.2%)
Total	393 (61.3%)	153 (23.8%)	72 (11.2%)	23 (3.5%)	641 (100%)	515 (80.3%)	212 (33.1%)	137 (21.4%)	110 (17.1%)	26 (4%)	1 (0.1%)

^a^ RO: radiographic ICDAS scoring of cFPMs.

### 3.1 Time from diagnosis to scheduled GA extraction

Ten months and 5 days was identified as the mean time between the initial assessment and the scheduled date of the extraction in this patient cohort. Specifically, 43.8% (88) of the extraction cases had a period of at least 6 months between the two dates, whereas 28.9% (58) waited for at least a year. Moreover, from the group of children who were planned for the extraction of at least one cFPM, the date of extraction was not determined in twelve (6%) of these cases.

## 4 DISCUSSION

In this service evaluation, 80.7% of the children sampled were planned or admitted for GA extractions because their most defective cFPM was designated to be of a poor restorative prognosis due to caries and/or enamel defects (Figure 1). The presence of enamel defects, recorded in 76.6% of children's worst‐affected cFPMs, appears to be a significant factor in the judgement of ‘poor’ prognosis, especially when the enamel shows signs of PEB, triggering the selection of extraction as the favoured management pathway. The extent of the enamel defect at the worst‐affected element, however, was at an initial stage in at least 36.4% (73) of the extraction cases. The most severe radiographic scores (the ICDAS scores 5 and 6) accounted for extraction in less than half (47.8%) of the children who had undergone a GA to extract at least one cFPM (Table 4). Also noteworthy, 40.8% of the extraction cases received no previous treatment in any of their cFPMs. The average of 10 months of time lag between the initial assessment and the scheduled date of the extraction (to favour spontaneous migration of the second permanent molar) might be used to instigate preventive and minimally invasive restorative stabilisation to help inform clinical judgement and decision‐making.

The rationale behind the current UK management of cFPMs can be explained by the common interpretation of the orthodontic guidelines, which advocate extraction of cFPM at an age that would lead to mesial migration of the second permanent molar into the space of the extracted first molar. This is predicated by the belief that even if a cFPM is restorable, its long‐term prognosis is still likely to be poor.^15^ The invasiveness of this approach in the UK compared with international counterparts could be explained by a lack of perceived clear guidance on how best to restore cFPMs and judge their prognosis in children.^7^, ^8^, ^10^ Natural physiological space closure following the cFPM extraction, however, is not always guaranteed. A meta‐analysis showed that clinically satisfactory space closure was observed in 72% of maxillary molars, dropping to 48% in the mandible even when the extraction is performed at the optimal stage of dental development.^20^ Spontaneous space closure is more likely when an associated third molar has been detected radiographically.^21^ Nevertheless, the third molar may not be visible radiographically at the time of extraction planning. In addition, a 13% prevalence of third molar agenesis in the British population is expected^22^ and this may leave many patients with only one molar in the quadrant from early as 9 years of age. Finally, the loss of FPMs may affect the occlusion due to the important role played by FPMs of children aged 5‐14 years in arch integrity and mastication.^23^, ^24^, ^25^ This further emphasises the importance of the application of minimally invasive restorative interventions in young children with cFPMs and raises the need for clear prognostic determinants of cFPMs to avoid the subjectivity of judgement, which might lead to over‐ or under‐treatment.^8^

Considering modern minimally invasive (MI) restorative evidence, half of these extraction cases (52.3%) where radiographic lesions did not exceed the middle third of dentine (the ICDAS scores 0‐4), as shown in Table 3, could be restored. A clinical study has shown that sealants were 100% effective at 12 months of follow‐up and 98% effective over 44 months in managing caries with up to the ICDAS score 4.^26^ Others have shown that resin‐based sealants have a survival rate of 72% after 18 months in 6‐ to 8‐year‐old children with mild defects,^27^ direct resin composites have a success rate of around 60% in severely affected cFPMs and 70% in moderate defects, whereas conventional GIC restorations have approximately 40% success rate in moderate and severely affected cFPMs after 24 months.^28^ Furthermore, prefabricated stainless steel crowns (SSCs) showed a 94.7% success rate after 24 months in 6‐ to 14‐year‐old children with severe defects,^29^ whereas cast metal restorations or indirect resin composite restorations had success rates of 90% and 85.7%, respectively, in cFPMs from 8‐ to 13‐year‐old children after 36 months.^30^

Interestingly, from the total of 201 children who had at least one cFPM extraction, 40.8% received no previous treatment in any of their FPMs. According to the binary logistic regression analysis and odds ratio analysis, patients who had worst‐affected FPMs with the ICDAS 3 and 4 radiographic scores had more chance of receiving stabilising restorations before extraction, whereas patients who had the ICDAS scores 1 and 2 had a reduced chance of having their cFPMs temporised, even with fissure sealants. The results of the present study suggest that these children were probably not directed towards minimally invasive restorative interventions, especially because cFPMs with initial stages of radiolucency had lower chances to be restored/treated and the temporised/restored teeth were probably never reassessed before the extraction. In fact, it would be ideal to know whether these temporised teeth showed signs of failure. In the population investigated in this study, temporisation was probably advised only to treat pain or retain function until the decided time of extraction.

Young permanent teeth with hypomineralisation defects are more susceptible to the caries process, and these patients should be targeted with caries prevention^31^ and regular recall to review any development of post‐eruptive breakdown.^32^ Minimally invasive strategies preserve teeth and offer time and cost savings and an increased longevity of the restored tooth‐restoration complex.^33^, ^34^, ^35^, ^36^ Moreover, they are more child‐friendly, are anxiety‐provoking and are just as effective as traditional treatments.^6^, ^37^, ^38^ Recently, a longitudinal clinical study using minimally invasive strategies to treat 6‐ to 8‐year‐old children with MIH‐affected teeth with lesions reaching the inner third of dentine using glass hybrid restorations showed high clinical and radiographic success (96.8%) after a 2‐year follow‐up.^39^

From the result of this service evaluation, the average of 10 months between the initial assessment and the scheduled date of the extraction might be utilised to instigate preventive and minimally invasive restorative stabilisation and assess the prognosis of this management approach without missing the physiological ‘extraction window’, if still required in future. Moreover, this approach would provide the patients and parents/carers with a more informed choice of treatment options available. Furthermore, it is known that there is a significant reduction in the occlusal accumulation of biofilm in fully erupted FPMs compared with partially erupted ones,^40^, ^41^ which reduces caries susceptibility as the occlusion develops.^41^ For hypomineralisation defects, there seems to be the same trend, since after completed tooth eruption, the non‐affected cervical enamel is exposed and the progression of PEB is limited. The behavioural adherence of the patient will improve with age, in addition to the complete eruption of the affected tooth leading to definitive contact points,^42^ ultimately improving a long‐term overall prognosis.^43^ All of these factors encourage and support the application of prevention regimes along with provisional minimally invasive restoration of young compromised FPMs, to retain these teeth, symptomless and in function, until definitive restorative management can be provided.

General anaesthesia was the preferred method of pain control for children who underwent extraction of cFPMs, regardless of the number of teeth that were extracted. This finding differs from an earlier study, which found a statistically significant difference between the number of teeth extracted and the choice of anaesthetic.^2^ This might also suggest the discrepancies that are present in the UK about the interpretation of the guidelines and the challenges faced by clinicians to perform this often‐traumatic extraction in a young child. Also, the assumption of economic factors advocating extraction over retaining cFPMs in the long term plays a critical role in decision‐making. Economic evaluations are important in determining the most cost‐effective course of action that will maximise the health of the society while meeting the values and requirements of the individual patient.^44^ A recent health economic study evaluated the overall costs and effectiveness of retaining cFPMs compared with timely extraction in children. The results of this study have shown that retaining cFPM can be more cost‐effective than extraction with spontaneous closure of the gap. The relative cost in that study was influenced by the cost of extraction under GA, and extraction of one or two cFPMs under general anaesthesia was not cost‐effective.^45^

Among the strengths of this service evaluation was that it was performed in one of the largest referral centres for paediatric dental care in the UK. Data collection and recording were meticulously carried out. Rigorous radiographic scoring and calibration was accomplished to counterbalance the limitation of the recording of PEB from the records of uncalibrated clinicians. This service evaluation highlighted the management of cFPMs at both tooth and child levels within a specialist setting. The developed methodology establishes a standard method and framework to permit comparisons with other similar centres in the UK and globally. To the authors' knowledge, no previous published studies have investigated exclusively and in such depth the management of children with cFPMs in a hospital setting. Humphreys and Albadri (2020) investigated the management of molar‐incisor hypomineralisation (MIH)–affected teeth in children and found that of the 48 sampled children, 25 (52.1%) patients had extraction of at least one MIH‐affected FPM.^46^ Taylor et al (2019) used clinical vignettes to investigate the management of the poor prognosis of FPMs by GDPs and specialists in paediatric dentistry. They revealed that GDPs would prefer to restore cFPMs in comparison with specialists, who were more likely to extract such teeth.^7^

Although this study has been conducted in one of the largest referral centres for paediatric dental care in the UK, the findings from this study may not be generalisable, as they reflect practice from only one centre. A further limitation of this study was that new patient referrals were suspended temporarily at the time of the data capture because the level of referrals had resulted in breaches of NHS treatment targets. The diagnosis of MIH was based on the records of clinicians who had not been calibrated to score these conditions. As such, the presence of MIH may have been under‐reported. Sedation is likely to have been under‐reported since there was no specific arm in sampling confined to sedation patients. Phase I (NP and JOP cohorts) analysis, however, confirmed that inhalation sedation was used in a minority of patients in this centre. Despite this, the developed methodology and data could be used to allow comparisons with other similar centres in the UK and internationally. Further studies comparing international approaches for the management of cFPMs in children should be performed to investigate differences, benefits and outcomes of each approach.

Children who were referred with cFPMs at GSTT tended to undergo hospitalisation for extraction under general anaesthesia. For just over half of the extraction cases, the worst‐affected tooth could potentially be restored using minimally invasive strategies, thus avoiding hospital admission. Paediatric dental specialists and general dental practitioners would benefit from clear prognostic and MI restorative guidelines. GDPs need to be supported and trained to be able to undertake more minimally invasive treatments on these key teeth in general practice, paralleled with an access to specialist opinions when needed. Some paediatric treatments will still necessitate the use of general anaesthesia or inhalation sedation; thus, more specialists in paediatric dentistry must be trained to acknowledge the modern MI restorative strategies to improve their clinical judgement of the prognosis of cFPMs and to manage some of them conservatively where possible, within the minimal intervention oral healthcare delivery framework.

## CONFLICT OF INTEREST

The authors declare that they have no known competing financial interests or personal relationships that could have appeared to influence the work reported in this paper.

## AUTHOR CONTRIBUTIONS

R. AlKhalaf, A.A Neves, F. Warburton, M.T Hosey and A. Banerjee contributed to conception and design; acquired, analysed and interpreted the data; and drafted and critically revised the manuscript. All authors gave the final approval and agreed to be accountable for all aspects of the work.
